# Characterization of Polypropylene Modified by Blending Elastomer and Nano-Silica

**DOI:** 10.3390/ma11081321

**Published:** 2018-07-30

**Authors:** Xiaohong Chi, Lu Cheng, Wenfeng Liu, Xiaohong Zhang, Shengtao Li

**Affiliations:** 1 State Key Laboratory of Electrical Insulation and Power Equipment, Xi’an Jiaotong University, Xi’an 710049, China; lu.cheng@stu.xjtu.edu.cn (L.C.); liuwenfeng@mail.xjtu.edu.cn (W.L.); sli@mail.xjtu.edu.cn (S.L.); 2 Key Laboratory of Engineering Dielectric and Its Application, Ministry of Education, Harbin University of Science and Technology, Harbin 150080, China; x_hzhang2002@hrbust.edu.cn

**Keywords:** PP-based dielectric, mechanical properties, dielectric properties, elastomer blending, nano-silica filling

## Abstract

Polypropylene (PP) contains promising application prospects in thermoplastic cables for high voltage direct current (HVDC) power transmission because of its outstanding thermal and dielectric properties. However, the problem of poor toughness and space charge has restricted the application of pure PP in HVDC cables. In this paper, polyolefin elastomer (POE) and nano-silica were blended thoroughly and added into a PP mixture by a melting method. Scanning electron microscopy (SEM) was employed to observe the dispersion of POE and nanoparticles. Thermal properties were characterized by differential scanning calorimetry (DSC) and dynamic mechanical analysis (DMA). Mechanical properties were evaluated by tensile tests. The elastomeric properties of composites were improved as the dispersed POE could transfer and homogenize external mechanical forces. DC breakdown results showed that the fail strength of composite with 10 phr POE and 1 phr nano-silica was obviously enhanced. The pulsed electro-acoustic (PEA) results showed that the injection and accumulation of space charge was increased by the introduction of POE, while it was restrained by the collective effect caused by nano-silica filling. X-ray diffraction (XRD) spectrograms showed that secondary ordered structures existed in the composites of PP, POE, and nano-silica, and that the ordered structure around the nanoparticles contributed to the enhancement of breakdown strength. The mechanical and dielectric properties were modified synergistically, which made the modified PP a propitious insulation material for HVDC cables.

## 1. Introduction

The high-voltage direct current (HVDC) system, which requires smaller cables and less loops, has obvious advantages over alter current (AC) systems in long-distance transmission. Nowadays, the HVDC system has become an important part of the electrical grid. Thus, more reliable and environment-friendly DC cables are required. Cross-linked polyethylene (XLPE) is the currently used insulation material for HVDC cables. However, XLPE is difficult to recycle due to its thermosetting structure. Besides, the problem of space charge caused by HVDC fields makes XLPE less dependable and greatly shortens its service life [1,2,3]. Unlike XLPE, polypropylene (PP) is easy to process and recycle because of its thermoplastic structure. Therefore, modified PP, which has excellent dielectric and thermo-mechanical properties, is a relatively ideal substitute for XLPE [4].

The accumulation of space charge under a HVDC field and its poor toughness limits the use of pure PP in HVDC cables. In order to improve the mechanical properties, elastomers, such as ethylene-propylene copolymer, and ethylene-octene copolymer (a kind of POE with a low dielectric constant) are blended with PP. The mechanical properties, such as toughness, impact, and tensile strength, are modified in the blends prepared by physical blending, and the application practicability of the blends in HVDC cables is under discussion [5,6,7,8,9]. However, the problem of space charge is more serious in blends of PP and POE [10]. The reliability deteriorates and service life is shortened by trapping, detrapping, agglomeration, and migration of space charge [11,12]. An effective method to repress space charge is to control trap distribution by forming the interphase of nano-filler and polymer matrix. Previous studies have shown that the introduction of light nano-fillers, such as MgO, Al_2_O_3_, silica (SiO_2_), montmorillonite (MMT), ZnO and so on, into semicrystalline polymers of PP or polyethylene (PE), successfully repress space charge and increase the breakdown strength [13,14,15,16]. The nano-SiO_2_ can disperse uniformly in PP and modify its thermal and mechanical properties, and the potential use of nanocomposites as an insulating material in HVDC cables has been proved [17,18].

In this paper, the POE and nano-SiO_2_ were blended into PP to modify its mechanical and dielectric properties. The growth of PP spherulites was inhibited by POE. The dispersed POE in PP, which can concentrate and transform mechanical stress, ameliorated the toughness of the sample. The function of nano-fillers in polymer matrix had two aspects. On the one hand, as the center of heterogeneous nucleation, it promoted the formation of micro crystals. On the other hand, it had a nanometer effect, which controlled the state structure of the composite, to suppress the space charge and improve the breakdown strength. Therefore, POE and nano-SiO_2_ particles were blended into the PP matrix by a melting method. This influenced the crystal structure of PP, and thus improved its mechanical and dielectric properties.

## 2. Materials and Experimental Procedure

### 2.1. Samples Preparation

The matrix was isotactic PP (HC314BF, Borealis, Abu Dhabi, United Arab Emirates), and its melt index was 3.9 g/10 min (230 °C/2.16 kg). The POE selected in this study was ethylene-octene copolymer particles (POE-0201, Exxon Mobil Corporation, Shanghai, China). Its melt index was 2.5 g/10 min (190 °C/2.16 kg), and its melting temperature was 97 °C. The content of octane in the copolymer was 20% to 30%, and its density was 0.902 g/cm^3^. The nano-SiO_2_ was modified by silica agent KH570, and the average diameter was 30 nm. The composite compound of PP and POE was melted using an internal mixer (HAAKE Polylab QC, torque rheometer, Waltham, MA, USA) at 180 °C, counter-rotating at a speed of 40 rpm for 20 min. In order to disperse the nano-filler, the nano-SiO_2_ and POE were first melted at 175 °C and 40 rpm for 15 min to prepare the master batch, then the PP was blended with the master batch at 180 °C and 40 rpm for 20 min to prepare the composite of PP/POE/SiO_2_. The 0.3 phr antioxidant (Irganox 1010, BASF, Shanghai, China) was mixed into all samples at the beginning of melting to avoid degradation. The antioxidant was organic powder and it could be melted into the polymer at the process temperature. The abbreviations and components of the composites are shown in Table 1. All of the samples were pressed to the required thickness with compression molding under 190 °C for 15 min.

### 2.2. Dispersion of POE and Nano-Silica

Different from PP, the POE could be dissolved in heptane. After immersion in heptane at 50 °C for 4 h, the POE dissolved and formed cracks at its surface. In an etch agent of potassium permanganate and concentrated sulfuric acid, POE and an amorphous fraction of PP were etched first, and the inner nano-silica was exposed. The PP/POE/SiO_2_ samples were etched in etch agent for 8 h at room temperature before observation. The samples after etching were sputtered by gold and observed by scanning electron microscope (SEM, KEYENCEVE-9800S, KEYENCE, Osaka, Japan).

### 2.3. Electrical Properties

According to the standard of ASTM D1389, the thickness of the samples for the DC breakdown experiment was about 0.13 mm. Tests were taken with a sphere-sphere electrode in insulating oil, and the voltage ramp was 2 kV/s. Breakdown tests were taken at least 30 times for each composition, and the Weibull statistic was used for analyzing their breakdown properties. The space charge distribution was measured by the pulsed electro-acoustic (PEA, Five Lab Peanut) method. The injection DC field was 60 kV/mm. The amplitude and pulse width of the pulse voltage were 600 V and 5 ns respectively. The voltage application time was 1800 s and the short circle time was 600 s.

### 2.4. Thermal and Mechanical Properties

Differential scanning calorimetry (DSC, STARe DSC822e, METTLER TOLEDO, Zurich, Switzerland) was employed to measure the melting and crystalline process. The melting and crystalline curves were gained from 25 to 190 °C with a 10 °C/min heating and cooling ramp. The crystallinity *W_c_* was calculated from the DSC result by Equation (1).

(1) $$W_{c}=\frac{∆H_{m}}{H_{0}\left(1-x\right)}\times 100\%$$

where, Δ*H_m_* was the melting enthalpy; *H*_0_ was the theoretical melting enthalpy of the completely crystallized form, and *H*_0_ was 209 J/g for the isotactic PP [19]; *x* was mass fraction of the inorganic fillers in composite.

The thermal-mechanical properties, such as loss modulus and storage modulus, were measured by 0.1 Hz mechanical vibrations over the temperature range from −30 to 100 °C with a dynamic mechanical analysis (DMA, METTLER SDTA1+, METTLER TOLEDO, Zurich, Switzerland) method. The measurement was taken with a tensile model at a heating rate of 2 °C/min, and the sample size was 0.1 mm × 4 mm × 9 mm. The mechanical tensile test was carried out by an electronic universal testing machine (CMT4304, METTLER TOLEDO, Zurich, Switzerland) according to the experimental standard of ISO 527-3:1995. The experimental sample was a 4 mm × 0.15 mm dumbbell sample with a measurement length of 20 mm, and the tensile rate was 100 mm/min. At least five samples of each group were measured to obtain an average value.

## 3. Results

### 3.1. Dispersion of POE and Nano-Filler

**Figure 1:**
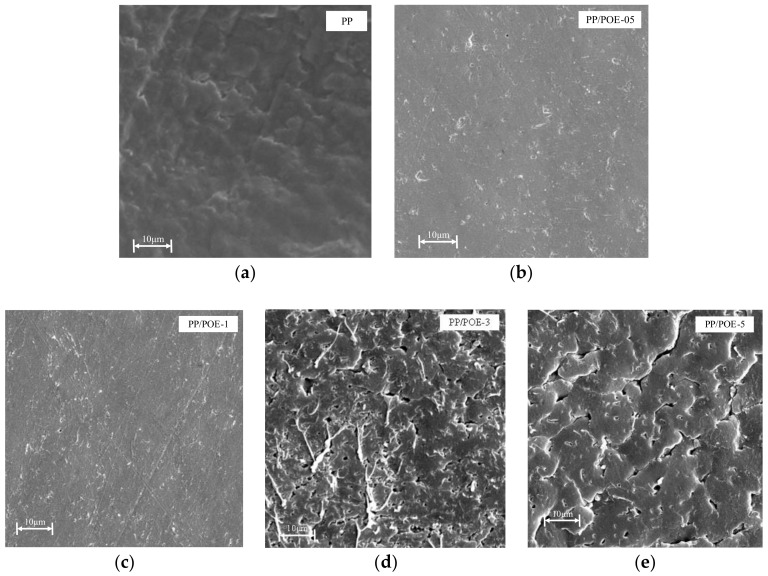
Dispersion of POE in the PP matrix. The micro cracks at surface were POE that were dissolved by heptane, and the size of the crack increased with the POE content: (**a**) PP; (**b**) PP/POE-05, (**c**) PP/POE-1, (**d**) PP/POE-3, (**e**) PP/POE-5.

The SEM images of composites with various POE content were shown in Figure 1. The micro-crack at the surface resulted from POE dissolving in heptane; thus, the size and distribution of POE could be indicated by that of the micro-cracks. Cracks did not form on the PP surface, as the PP could not dissolve in heptane. POE was dispersed in the PP matrix and the size was smaller than 1 μm in a composite with low content, as shown in images of PP/POE-05 and PP/POE-1. The size of the crack was increased, with an increase of POE content, which formed a continuous distribution. The size of POE was larger than 10 μm in samples of PP/POE-3 and PP/POE-5.

**Figure 2:**
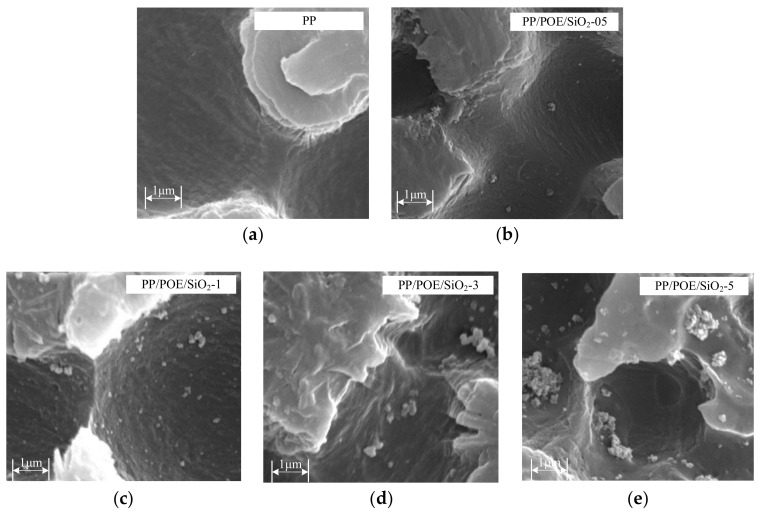
Dispersion of nano-SiO_2_ particles in the nanocomposites. The high brightness and continuous regions were PP crystalline areas that were not attacked in the etching process, and the small spherical regions were nano-SiO_2_: (**a**) PP, (**b**) PP/POE/SiO_2_-05, (**c**) PP/POE/SiO_2_-1, (**d**) PP/POE/SiO_2_-3, (**e**) PP/POE/SiO_2_-5.

The nano-silica was exposed after POE, and the amorphous PP was etched by a solution of potassium permanganate and concentrated sulfuric acid. The high brightness and continuous regions in the images indicated the PP crystal areas that were not corroded, and the small spherical regions were nano-silica, as shown in Figure 2. The nano-silica was dispersed evenly in the composites of PP/POE/SiO_2_-05 and PP/POE/SiO_2_-1, and its size was about 100 nm. The agglomeration emerged as the content of nano-silica increased. The nano-silica aggregated together and formed a micron-scale distribution, as shown in the images of PP/POE-3 and PP/POE-5.

### 3.2. DC Breakdown Strength

The breakdown field strength was an important factor to evaluate the reliability of the insulation. In this study, the DC breakdown strength of PP and the composites with different concentrations of POE and nano-silica were measured. Dielectric breakdown followed a certain probability distribution. The Weibull distribution was used to analyze the breakdown data. The probability distribution function of breakdown strength could be expressed by Equation (2):

(2) $$P_{f}\left(E_{b};\alpha ,\beta \right)=1-exp\left\{-{\left(\frac{E_{b}}{\alpha }\right)}^{\beta }\right\}$$

where: *E_b_* was the experimental value of the breakdown strength (kV/mm). *α* was the strength of 63.2% breakdown probability, which was known as the scale parameter and the characteristic breakdown field strength (kV/mm). *β* was a shape parameter that represented the probability distribution of breakdown field strength, representing the dispersion of the breakdown data. *P_f_* was the probability distribution function. For 30 samples, it could be expressed as a probability distribution Function (3):

(3) $$P_{f}\left(i,n\right)=\frac{i-0.3}{n+0.4}$$

where: *i* was the sample sequence number and *n* were the sample numbers.

**Figure 3:**
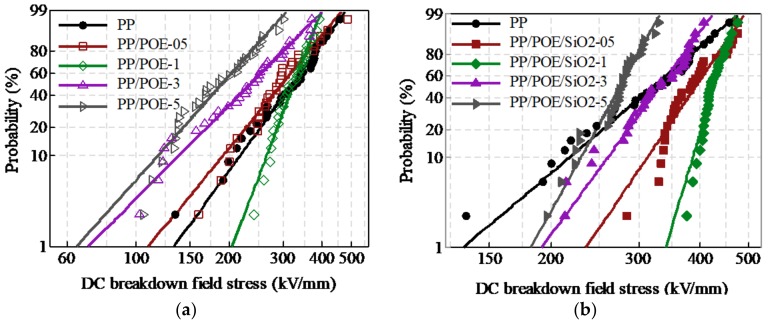
Weibull distribution of breakdown strength. (**a**) was the electric strength distribution of PP with POE blending, (**b**) was the electric strength distribution of PP with POE and nano-SiO_2_ blending.

The statistical distribution of 30 samples of breakdown strength in each group were analyzed by Weibull statistics, as shown in Figure 3, and the Weibull statistic data is listed in Table 2. The breakdown strength was reduced with an increase in POE content, as shown in Figure 3a. The shape parameter *β* of PP/POE-1 with 10 phr POE content was increased, which indicated that the scatter of breakdown strength decreased. This was caused by the connection of POE. The continuous distribution of large-scale POE formed weak areas for electric field concentration, where electrons moved easily and resulted in breakdown. The filling of nano-silica enhanced the breakdown strength, as shown in Figure 3b.

With an increase nano-silica content, the breakdown strength of composite was first increased, and then it decreased. The maximum value was reached when the composite contained 1 phr nano-silica and 10 phr POE. In the samples of PP/POE/SiO_2_-1 with 1 phr nano-silica and 10 phr POE, the α increased by 25% and β was raised by about four-fold. The results of the DC breakdown test showed that the PP/POE-1 and PP/POE/SiO_2_-1 could be selected as potential insulation materials for HVDC cables, and so the space charge, thermal, and mechanical properties of this two samples were tested and analyzed.

### 3.3. Space Charge Distribution

**Figure 4:**
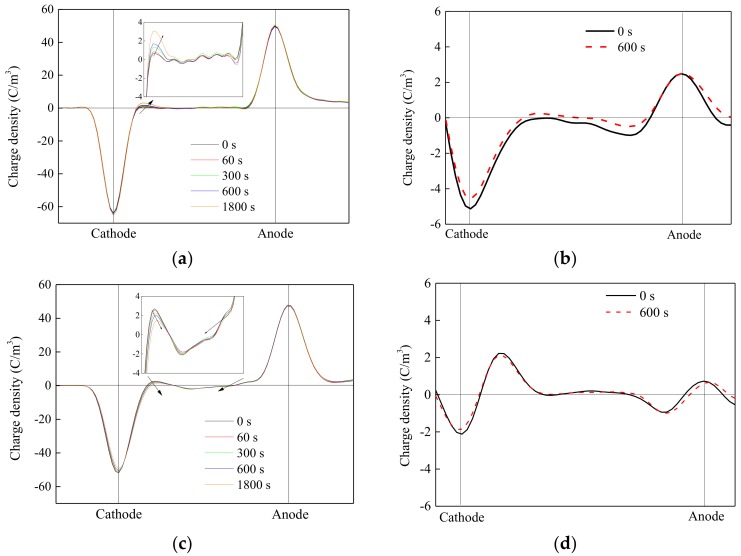

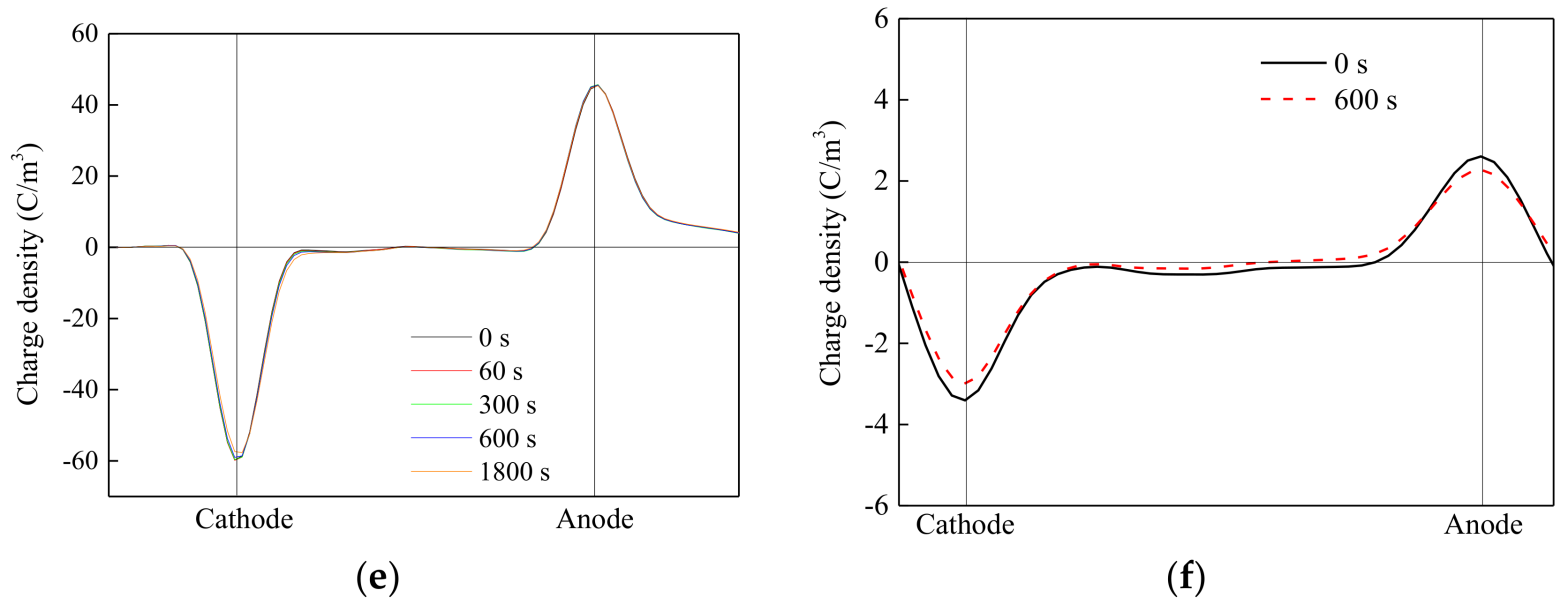
Space charge distribution at a strength of 60 kV/mm and short circuit. (**a**,**b**) was the space charge distribution of PP with voltage applied and in a short circuit, respectively. (**c**,**d**) was the space charge distribution of PP with a 10 phr POE content, with voltage applied and in a short circuit, respectively. (**e**,**f**) was the space charge distribution of PP with 10 phr POE and 1 phr nano-SiO_2_ content with voltage applied and in a short circuit, respectively.

The injection, accumulation, and migration of space charge could result in insulation aging and failure. Therefore, it was important to suppress the space charge in the DC insulation design. The space charge measurement was taken with the samples of PP/POE/SiO_2_-1 and PP/POE-1, which had obvious rises in breakdown strength. The distribution of space charge in 60 kV/mm DC stress is shown in Figure 4. The DC voltage was applied for 1800 s and sampled at 0 s, 60 s, 300 s, 600 s, and 1800 s, and then short circuited for 600 s.

Under DC stress, the hetero-charges near the cathode in the PP sample accumulated, and the amount of charges increased with an increase in the voltage0applied time, and more charges moved to the interior. A small amount of hetero-charges were retained near the anode, and they were attenuated at the short circuit time, as shown in Figure 4a,b. Hetero-charges accumulated near the cathode and homo-charges appeared near the anode in the PP/POE-1 sample. The hetero-charge moved into bulk under the application of voltage. After short circuit, the hetero-charge accumulated in the vicinity of the cathode and anode, and residual charges in the sample attenuated slowly with time, as shown in Figure 4c,d. Impurities and weak-bound ions in the PP and blends, which originated from raw materials and manufacturing process, could be polarized or ionized under a DC electric field. Meanwhile, charges would be injected from the electrode into the samples. The charges originating from the above sources could be trapped, and then space charge accumulation would take place [20]. Since the positive ion was heavier than the electron, the mobility of the positive ion was much lower than that of the electron under the same applied stress. Thus, the positive space charge accumulation formed near the cathode in the pure PP and PP/POE-1 [10]. There was almost no charge accumulated in PP/POE/SiO_2_-1 under DC stress. Furthermore, the residual charges were of a small number and almost completely attenuated after short circuit for 600 s, as shown in Figure 4e,f. Nano-silica fillings, which inhibited the injection of space charge, was responsible for this difference.

### 3.4. Thermal Properties

**Figure 5:**
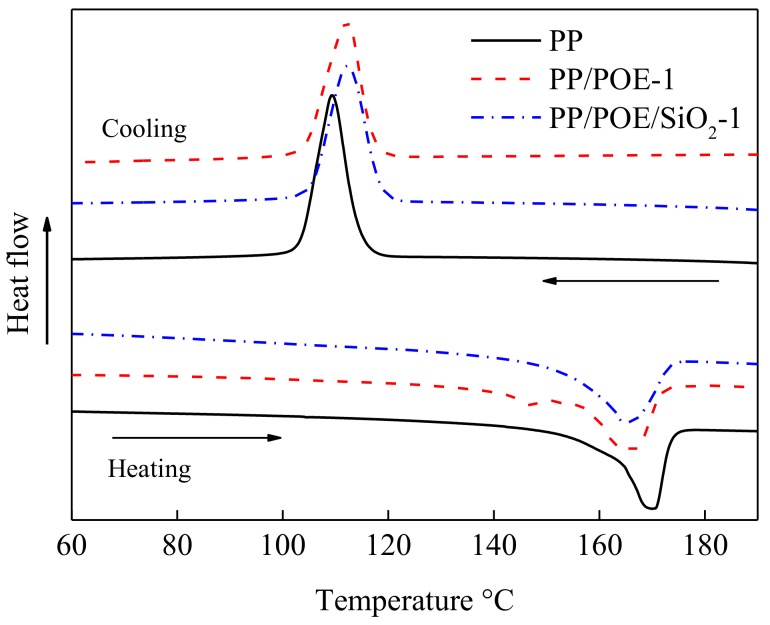
Heating and cooling curves of DSC.

The enthalpy curves of heating and cooling of DSC were shown in Figure 5. The melting enthalpy Δ*H_m_* and melting temperature *T_m_* could be calculated by heating curves. The crystalline onset temperature *θ*_0_, crystallization peak temperature *θ_p_* and half width of crystalline peak Δ*W* could be gained from cooling curves.

The parameters of melting and crystallizing were listed in Table 3. The crystalline onset temperature *θ*_0_ and crystallization peak temperature *θ_p_* of PP/POE/SiO_2_-1 were higher than PP, which indicated that the filling of POE and the nano-silica promoted crystallization at high temperature. Because the POE blocked the growth of crystal, the crystallinity of PP/POE-1 was lower than that of the PP, while the Δ*W* increased. The melting temperature *T_m_* of composites was lower than that of PP because of the introduction of POE, which had a low crystallinity and melting temperature. Endothermic melting occurred below 150 °C in PP/POE-1, which was attributed to the faster activation of the POE chain than the PP chain in the heating process. Crystallinity was related to the cooling process. In order to reveal real crystallization, the value of crystallinity was calculated from the melting integral value. The process of heterogeneous nucleation, enhanced by nano-silica filling, promoted even growth of small crystals in PP/POE/SiO_2_-1.This was the reason for the increase in crystallinity.

### 3.5. XRD Analysis

**Figure 6:**
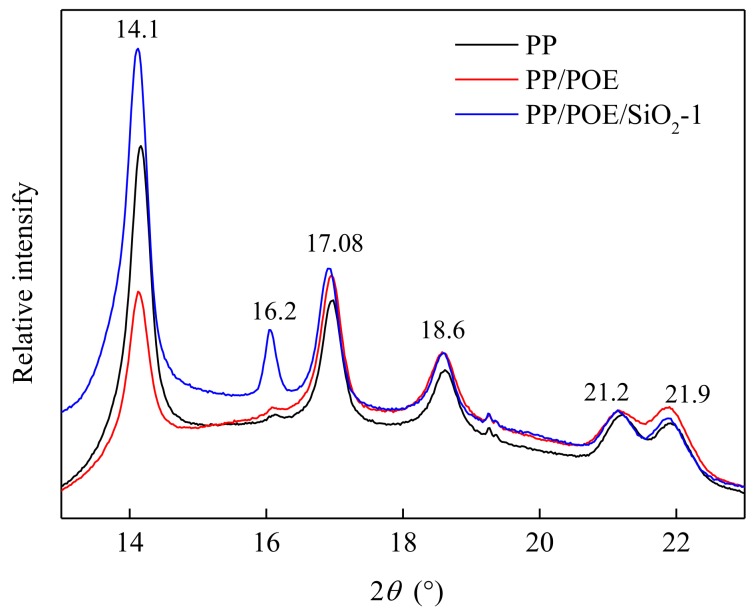
X-ray diffraction (XRD) spectra of PP and composites at ambient temperature.

The intensity of X ray diffraction (XRD) is shown in Figure 6. The peaks of PP/POE/SiO_2_-1 had the highest intensity, which indicated that the crystallinity of PP/POE/SiO_2_-1 was higher than that of PP and PP/POE, consistent with the results of DSC. The diffraction peak at a Bragg angle of 14.1°, 17.08°, and 18.6° was the surface diffraction of a monoclinic crystal (α crystal), which indicated that the main crystal structures of those samples were α crystals [21].

The low split peaks at 21.2° and 21.9° were caused by the diffraction of incomplete crystallization. In PP/POE-1, the intensity of split peaks was the highest, because there was more incomplete crystallization in this sample than the others. The filling of nano-SiO_2_ promoted the formation of hexagonal crystals (β crystal), which were proven by the diffraction peak of the β crystal appearing at 16.2° in PP/POE/SiO_2_-1.

### 3.6. Thermal-Mechanical Properties

**Figure 7:**
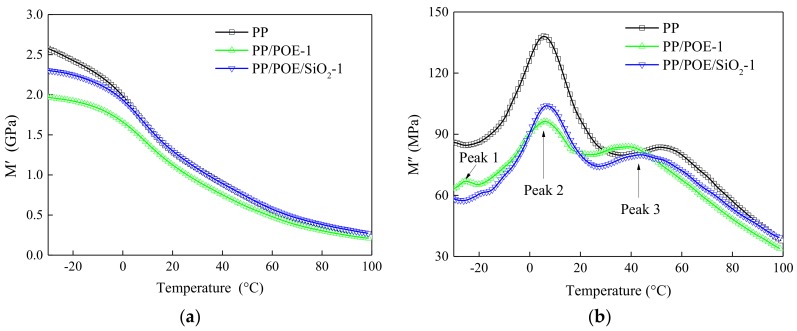
Storage modulus and loss modulus of PP and composites measured by dynamic mechanical analysis (DMA). (**a**) The storage modulus. (**b**) The loss modulus.

The storage modulus M′ and loss modulus M″ of PP/POE-1 and PP/POE/SiO_2_-1 was shown in Figure 7. When the temperatures were below 0 °C, the storage modulus M′ of all samples was higher than 1 GPa. This indicated that the PP and composites were rigid at this temperature. The storage modulus M' and integral of loss peak 2 of PP/POE-1 and PP/POE/SiO_2_-1 were lower than PP, which was attributed to the increase of elasticity caused by POE blending. The POE had elastic behavior and low loss modulus. The molecular chain was easy to move under external force. The POE dispersed in PP could transfer and homogenize the external force and increase the elasticity.

Peak 2 and peak 3 in Figure 7b appeared at a range of 20 to 60 °C and −10 to 10 °C respectively, which were attributed to different phase changes. The loss peak 2 represented an amorphous phase transition around the glass transition temperature. The loss peak 3 corresponded to a secondary ordered structure transition in an amorphous phase, and the temperature of secondary transition was higher than that of the amorphous phase [22]. The loss peak 1 at −20 °C was from the phase transmission of POE that was linked weakly with PP in PP/POE-1 samples. The POE promoted the secondary phase moving to lower temperatures in the composites. That was why the peak 3 temperature of PP/POE-1 and PP/POE/SiO_2_-1 was lower than PP.

### 3.7. Mechanical Properties

**Figure 8:**
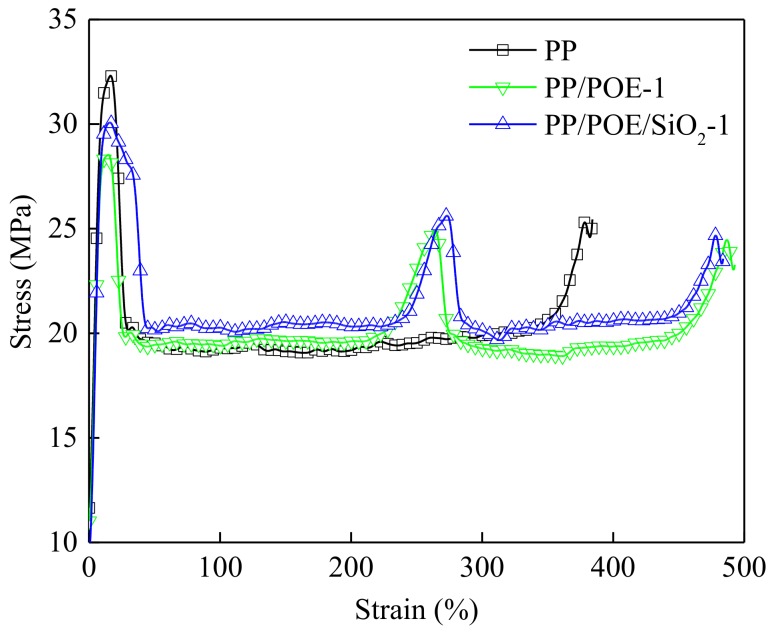
Tensile stress-strain curves of PP and composites.

Mechanical properties are one of the important factors that hinder the application of PP in cables. In order to evaluate the application feasibility of PP-based composite in HVDC cables, a mechanical tensile test was carried out. The stress-strain curve was shown in Figure 8. The long octyl chain in the POE molecular structure formed a joint point, which could joint and buffer the applied stress. When the sample was subjected to tension, the network structure formed by these junction points could be greatly deformed and transfer the stress. Therefore, the elongation at break of the composite with POE had a significant increase.

The yield strain points of composites with POE lagged behind PP, so that second stress concentrations appeared between 200% and 300% tensile strain in the composites. The inorganic nano-SiO_2_ particle was rigid in itself, and the heterogeneous nucleation of the nanoparticles could improve the crystallinity of the composite. Two aspects mentioned above may have reduced the toughness. In this study, the filling of nano-SiO_2_ particles had little effect on the toughness of composite. The main reason for this was that the POE molecular chain around the nano-SiO_2_ particles formed a non-rigid filling, and at the same time the nano-scale-dispersed nano-SiO_2_ particles formed a strong interaction with the polymer chain. The mechanical tensile parameters of PP and its composites are shown in Table 4. The blending of PP/POE reduced the yield strength and elastic modulus of the composites, consistent with the results of DMA. The decrease of elastic modulus showed that the stiffness of the composites decreased and the toughness increased, which was conducive to the application in cable insulation. Comparing the stress-strain characteristics of PP/POE-1 and PP/POE/SiO_2_, it could be seen that the addition of nanoparticles had little effect on the mechanical properties.

## 4. Discussion

**Figure 9:**
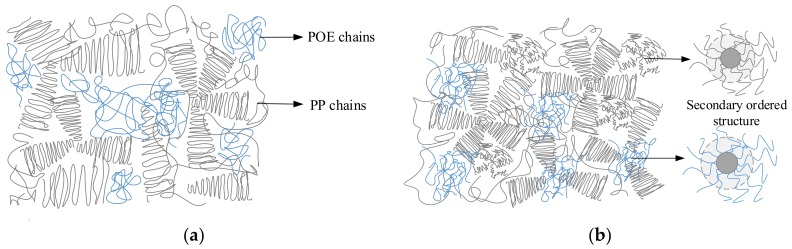
Arrangement of molecule chain of composites. (**a**) Diagram of the molecule chain of PP blended with POE. (**b**) Diagram of molecule chain of PP blended with POE and nano-SiO_2_.

In the PP/POE-1 composite, POE was mainly dispersed in the matrix and the low order molecular chain was related to amorphous phase of PP, as shown in Figure 9a. The distribution of POE in PP hindered the orderly arrangement of chains, and thus hindered the crystallization of PP, leading to a decrease in the crystallinity of the PP/POE-1. The secondary phase change temperature in the amorphous region was also reduced by POE, which showed the decrease of the α phase change temperature in loss modulus of DMA results. In the blend of PP and POE, the main interaction was physical and not completely compatible. The disordered chains of POE were activated faster than the PP chains during heating, appearing as a γ phase change in the XRD results, and resulting in endothermic melting below 150 °C in the DSC results [8]. A large distributed localized state introduced by POE promoted the injection and accumulation of the space charge. Therefore, a number of holes accumulated in the PP/POE composite. The weak relation of POE between the PP matrix formed structure defects, which increased the free length and promoted the energy storage of the carrier. The stress concentration caused by the space charge and the carrier energy accumulated in the defects, which resulted in the breakdown of the PP/POE composite, and the breakdown strength decreased with an increase of POE content.

The heterogenous nucleation of nano-SiO_2_ improved the order arrangement of the polymer chain. The PP and POE chain were arranged radially around the nano-SiO_2_ and formed new and compact secondary ordered structures in the amorphous region [23], as sketched in Figure 9b. The crystallinity of PP/POE/SiO_2_-1 and the interaction of PP and POE was enhanced by the secondary structure around nano-SiO_2_, and the movement of the chain was restricted. Thus, only one peak of endothermic melting was formed in the DSC results, and the temperature of the α phase change increased. At the same time, the ordered structure around the nano-SiO_2_-introduced localized state suppressed the injection of the space charge, especially the hole [24,25]. The improvement of structural integrity and inhibition of space charges enhanced the breakdown strength of PP/POE/SiO_2_-1 composites.

## 5. Conclusions

The PP-based composites with various POE and nano-SiO_2_ content were fabricated by a melting blend method, and samples were evaluated by DC breakdown strength to select the optimum composition. The difference in chain arrangement was revealed by thermal and thermal-mechanical properties indirectly.

In composites compounded with PP and POE, a micro-scale continuous phases formed when the POE content was more than 10 phr. In nanocomposites compounded with PP, POE, and nano-SiO_2_, agglomerations of nano-fillers emerged when their contents exceeded 1 phr. The large continuous phase of POE and aggregation of nano-SiO_2_ resulted in a decrease of breakdown strength.

In PP/POE-1 composites, the crystalline and storage modulus were reduced by the introduction of POE, which increased the feasibility of application in HVDC cables. Nevertheless, the amorphous POE were incompletely compatible with PP, and the dispersed phase inhibited the growth of crystals and formed structure defects, leading to the injection and accumulation of space charge and the reduction of breakdown strength.

In PP/POE/SiO_2_-1 composites, the modification of thermal and mechanical properties were hardly affected by nano-SiO_2_ doping, while the space charge was suppressed by the secondary ordered structure around nano-SiO_2_, which increased the DC breakdown strength. PP has broad application prospects in HVDC cables, with synergistically modified mechanical, thermal and dielectric properties.

## Figures and Tables

**Table 1:** Abbreviation and component of samples.

No.	Mass Fraction, phr		
	PP	POE	Nano-SiO_2_
PP	100	0	0
PP/POE-05	95	5	0
PP/POE-1	90	10	0
PP/POE-3	70	30	0
PP/POE-5	50	50	0
PP/POE/SiO_2_-05	95	5	0.5
PP/POE/SiO_2_-1	90	10	1
PP/POE/SiO_2_-3	70	30	3
PP/POE/SiO_2_-5	50	50	5

**Table 2:** Weibull statistic data of breakdown strength.

Samples	Shape β	Scale α (kV/mm)
PP	4.801	345.6
PP/POE-05	4.252	323.2
PP/POE-1	9.206	338.0
PP/POE-3	3.502	259.8
PP/POE-5	3.907	207.9
PP/POE/SiO_2_-05	8.310	408.1
PP/POE/SiO_2_-1	18.780	434.6
PP/POE/SiO_2_-3	7.717	346.5
PP/POE/SiO_2_-5	10.250	284.9

**Table 3:** Parameters of isothermal crystallization and the melting process.

Parameters of Melting and Crystallizing	PP	PP/POE-1	PP/POE/SiO_2_-1
Melting temperature *T_m_*/°C	161.5	152.3	157.8
Crystalline onset temperature *θ*_0_/°C	114.9	117.5	119.2
Crystallization peak temperature *θ_p_*/°C	109.4	112.1	112.3
Half width of crystalline peak Δ*W*/C	3.2	3.7	3.7
Integrals of crystallization exothermic peak/J/g	107.9	100.7	115.2
Integrals of melting endothermic peak/J/g	92.9	82.1	98.4
Crystallinity *W_c_*/%	44.5	39.3	47.1

**Table 4:** Parameters of the tensile test.

Parameters of the Tensile Test	PP	PP/POE-1	PP/POE/SiO_2_-1
Modulus/MPa	1524 ± 46	1361 ± 28	1465 ± 37
Yield stress/MPa	32 ± 1.9	28 ± 1.1	30 ± 1.1
Strain at break/%	379 ± 23	493 ± 10	489 ± 9.8
Yield stress near 275% strain/MPa	-	25 ± 1.3	26 ± 1.2

## References

[1] Stevens G.C., Freebody N.A., Hyde A., Perrot F., Szkoda-Giannaki I., Vaughan A.S., Medlam J.A. Balanced nanocomposite thermosetting materials for HVDC and AC applications. Proceedings of the 2015 IEEE Electrical Insulation Conference (EIC).

[2] Zhou F., Li J., Yan Z., Zhang X., Yang Y., Liu M., Min D., Li S. (2016). Investigation of charge trapping and detrapping dynamics in LDPE, HDPE and XLPE. IEEE Trans. Dielectr. Electr. Insul..

[3] Zha J., Yan H., Li W., Dang Z. (2016). Morphology and crystalline-phase-dependent electrical insulating properties in tailored polypropylene for HVDC cables. Appl. Phys. Lett..

[4] Dang B., He J., Hu J., Zhou Y. (2015). Tailored sPP/silica nanocomposite for ecofriendly insulation of extruded HVDC cable. J. Nanomater..

[5] Lin T.A., Lou C.-W., Lin J.-H. (2017). The Effects of thermoplastic polyurethane on the structure and mechanical properties of modified polypropylene blends. Appl. Sci..

[6] Geng C., Su J., Han S., Wang K., Fu Q. (2013). Hierarchical structure and unique impact behavior of polypropylene/ethylene-octene copolymer blends as obtained via dynamic packing injection molding. Polymer.

[7] Delhaye V., Clausen A.H., Moussy F., Hopperstad O.S., Othman R. (2010). Mechanical response and microstructure investigation of a mineral and rubber modified polypropylene. Polym. Test..

[8] Du H., Zhang Y., Liu H., Liu K., Jin M., Li X., Zhang J. (2014). Influence of phase morphology and crystalline structure on the toughness of rubber-toughened isotatic polypropylene blends. Polymer.

[9] Green C.D., Vaughan A.S., Stevens G.C., Pye A., Sutton S.J., Geussens T., Fairhurst M.J. (2015). Thermoplastic cable insulation comprising a blend of isotactic polypropylene and a propylene-ethylene copolymer. IEEE Trans. Dielectr. Electr. Insul..

[10] Zhou Y., He J., Hu J., Huang X., Jiang P. (2015). Evaluation of polypropylene/polyolefin elastomer blends for potential recyclable HVDC cable insulation applications. IEEE Trans Dielectr. Electr. Insul..

[11] Yu S., Li S., Feng Y. (2016). Progress in and prospects for electrical insulating materials. High Voltage.

[12] Zhang L., Zhang Y., Zhou Y., Teng C., Peng Z., Spinella S. (2018). Crystalline modification and its effects on dielectric breakdown strength and space charge behavior in isotactic polypropylene. Polymers.

[13] Wang Y., Wang C., Chen W., Xiao K. (2016). Effect of stretching on electrical properties of LDPE/MgO nanocomposites. IEEE Trans Dielectr. Electr. Insul..

[14] Lv Z., Wang X., Wu K., Chen X., Cheng Y., Dissado L.A. (2013). Dependence of charge accumulation on sample thickness in nano-SiO_2_ doped LDPE. IEEE Trans Dielectr. Electr. Insul..

[15] Chiu F.C., Yen H.Z., Lee C.E. (2010). Characterization of PP/HDPE blend-based nanocomposites using different maleated polyolefins as compatibilizers. Polym Test..

[16] Tian F., Lei Q., Wang X., Wang Y. (2012). Investigation of electrical properties of LDPE/ZnO nanocomposite dielectrics. IEEE Trans Dielectr. Electr. Insul..

[17] Pourrahimi A.M., Pallon L.K.H., Dongming Liu D.M., Hoang T.A., Gubanski S., Hedenqvist M.S., Olsson R.T., Gedde U. (2016). Polyethylene nanocomposites for the next generation of ultra-low transmission-loss HVDC cables: Insulations containing moisture-resistant MgO nanoparticles. ACS Appl. Mater. Interfaces.

[18] Bikiaris D.N., Papageorgiou G.Z., Pavlidou E., Vouroutzis N., Palatzoglou P., Karayannidis G.P. (2006). Preparation by melt mixing and characterization of isotactic polypropylene/SiO_2_ nanocomposites containing untreated and surface-treated nanoparticles. J. Appl. Polym. Sci..

[19] Radovanovic P.D., Krogseng G.P., Waller C.P., Mrozinski J.S., Krueger D.L. (1999). Temperature-Sensitive Microporous Film. United States Patent.

[20] Montanari G.C. (2010). Bringing an insulation to failure: The role of space charge. IEEE Trans Dielectr. Electr. Insul..

[21] Lv Z., Hu C., Xue J., Dou T. (2010). Effect of zeolite 5A on the crystalline behavior of polypropylene (PP) in PP/β-nucleating agent system. Polym. Compos..

[22] Shen J.B., Li J., Guo S.Y. (2012). The origin of a new transition in dynamic mechanical spectra of multilayer polymeric composite. Polymer.

[23] Kochetov R., Andritsch T., Morshuis P.H.F., Smit J.J. (2012). Anomalous behavior of the dielectric spectroscopy response of nanocomposites. IEEE Trans Dielectr. Electr. Insul..

[24] Wang W., Min D., Li S. (2016). Understanding the conduction and breakdown properties of polyethylene nanodielectrics: Effect of deep traps. IEEE Trans Dielectr. Electr. Insul..

[25] Li S., Yin G., Bai S., Li J. (2011). A new potential barrier model in epoxy resin nanodielectrics. IEEE Trans Dielectr. Electr. Insul..

